# Supplementary figures and images for: Experimental determination of the force of malaria infection reveals a non-linear relationship to mosquito sporozoite loads
Source: PLoS Pathog. 2020 May 26;16(5):e1008181. doi: 10.1371/journal.ppat.1008181 (PMC7295235; doi:10.1371/journal.ppat.1008181)

A

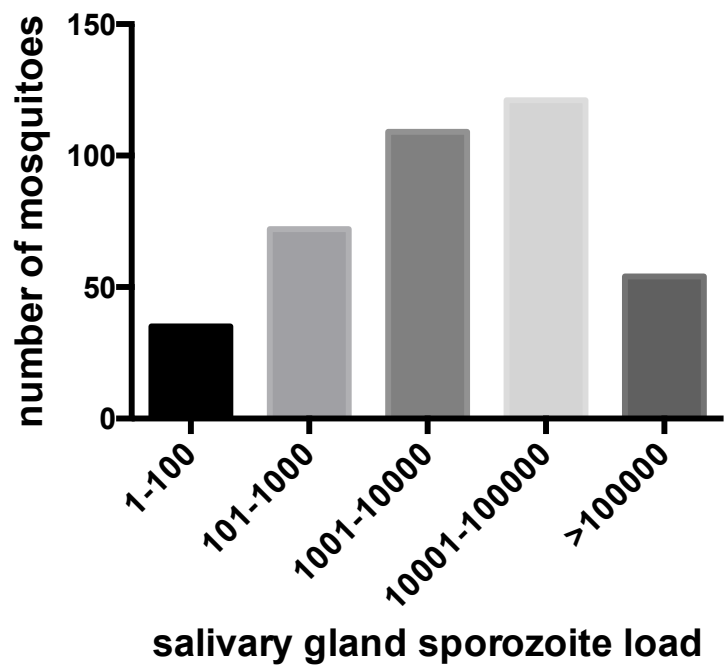

B

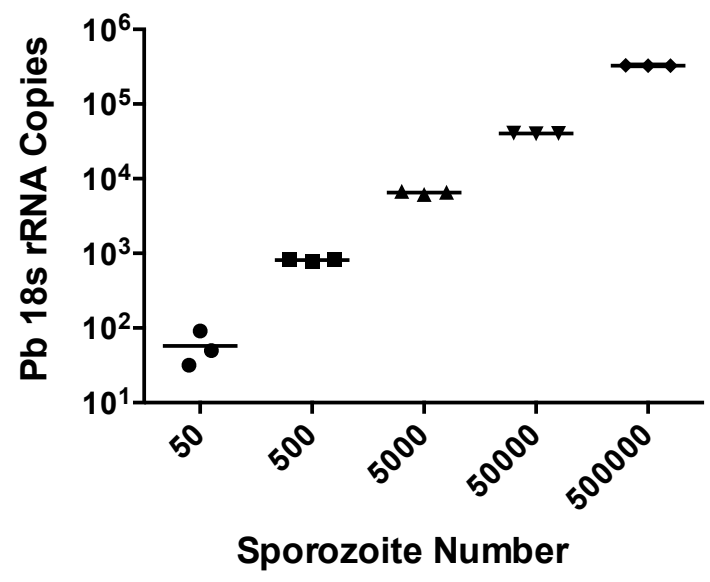

Supplement: S1 Fig — (A) Salivary gland loads are binned as indicated in the X-axis and shown is the number of mosquitoes that fall into each bin. The median salivary gland load is 8,865 sporozoites. 216 mosquitoes had <10,000 and 196 mosquitoes had >10,000 salivary gland sporozoites. (B) qPCR quantification of 10-fold dilutions of a known number of sporozoites using 18s rRNA plasmid copy numbers as a standard. (PDF) [file ppat.1008181.s001.pdf]

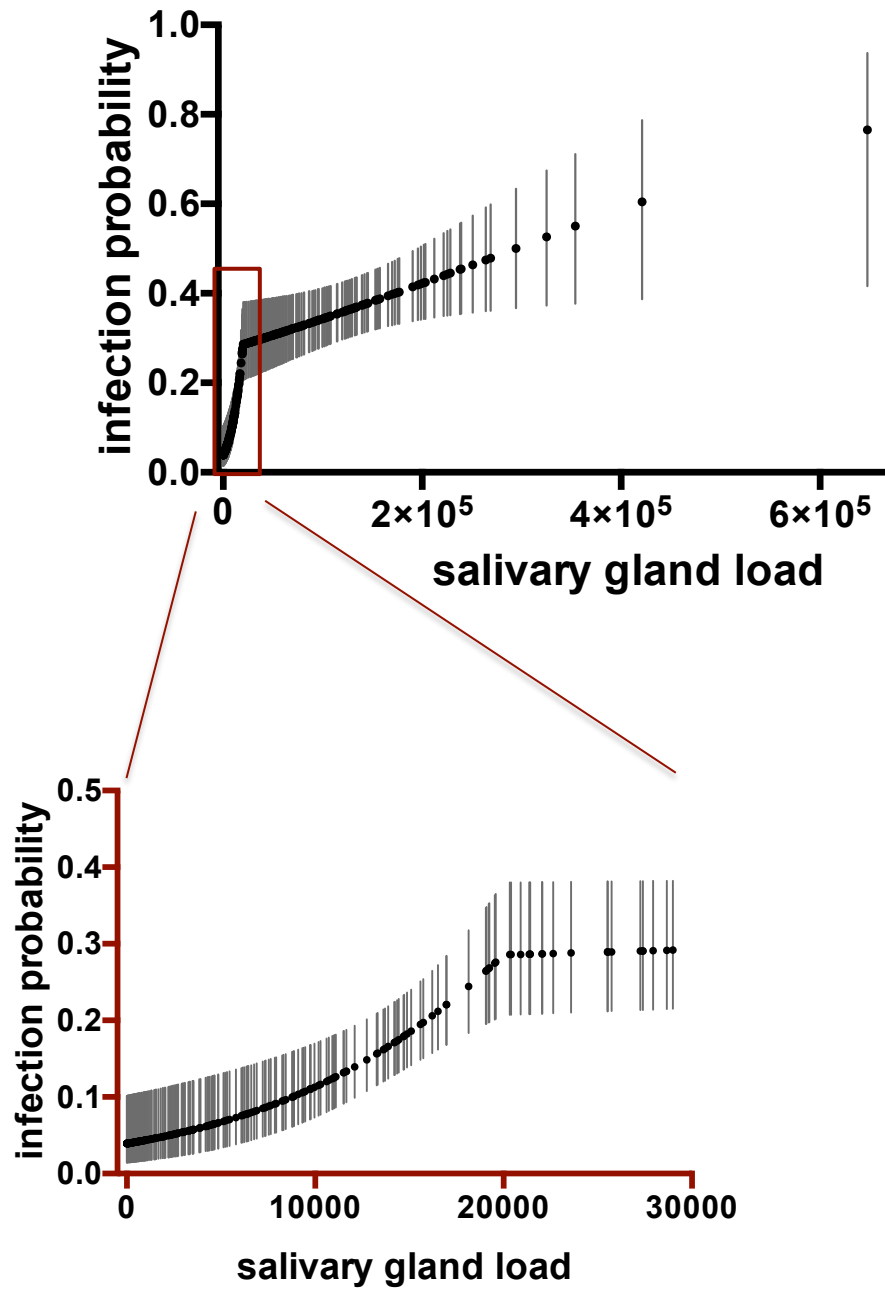

Supplement: S2 Fig — The full dataset, extending from salivary gland loads of 1 to 647,714 sporozoites, is plotted with 95% confidence intervals for each point. The graph below displays the data from mosquitoes with salivary gland loads of 1 to 30,000 (boxed region of top graph) to better display the data in this range. (PDF) [file ppat.1008181.s002.pdf]

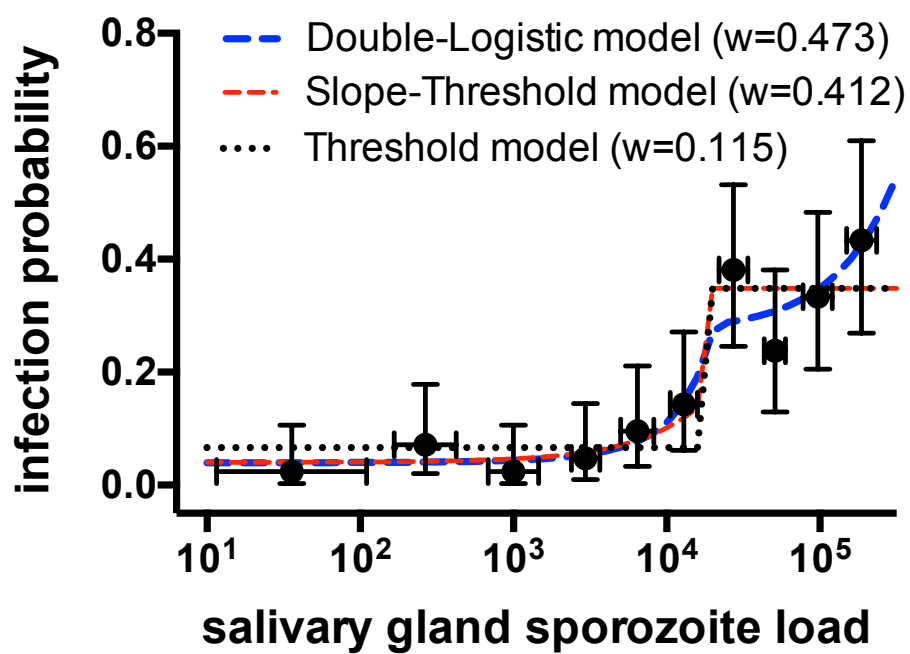

Supplement: S3 Fig — We fit three different mathematical models that include a rapid increase in infection probability (“threshold”, “slope-threshold”, or “double-logistic”) to the mouse-mosquito dataset (n = 408 mouse-mosquito pairs with salivary gland loads between 1 and 300,000 sporozoites), using the maximum likelihood method (see Eq 6 in Materials and Methods). The quality of the model fit to data was calculated using Akaike Information Criterion to generate Akaike weights (w). While the models incorporating a gradual rise between salivary gland loads of 10,000 to 20,000 sporozoites provided a better fit based on w values, the differences between the weights was small and the Hosmer-Lemeshow goodness of fit test indicated that all 3 models fit the data well (p>0.1). The threshold, slope-threshold, and double-logistic models are described by Eqs 3–5, respectively, in the Materials and Methods. Parameters of the models providing the best fit are also given in the Materials and Methods. (PDF) [file ppat.1008181.s003.pdf]

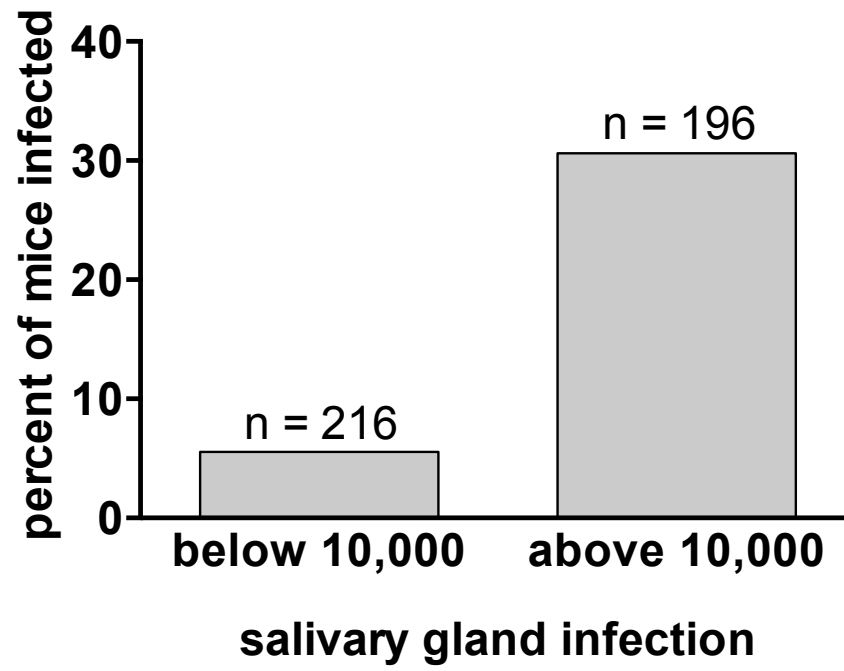

Supplement: S4 Fig — Single-mosquito feeds were performed, and subsequently salivary gland sporozoite loads were measured and mice were followed for blood stage malaria infection. Mosquitoes are binned as to whether they had greater than or less than 10,000 salivary gland sporozoites, and the probability that the mosquitoes in each group will initiate a malaria infection is plotted. Salivary gland sporozoite load is significantly associated with the likelihood of malaria infection (odds ratio 7.5, CI 3.6–15.8, p<0.001). n = total number of mouse-mosquito pairs in each bin, pooled from 20 independent experiments with 7 to 44 mouse-mosquito pairs per experiment, with total n = 412. (PDF) [file ppat.1008181.s004.pdf]

Supplementary Figure 5

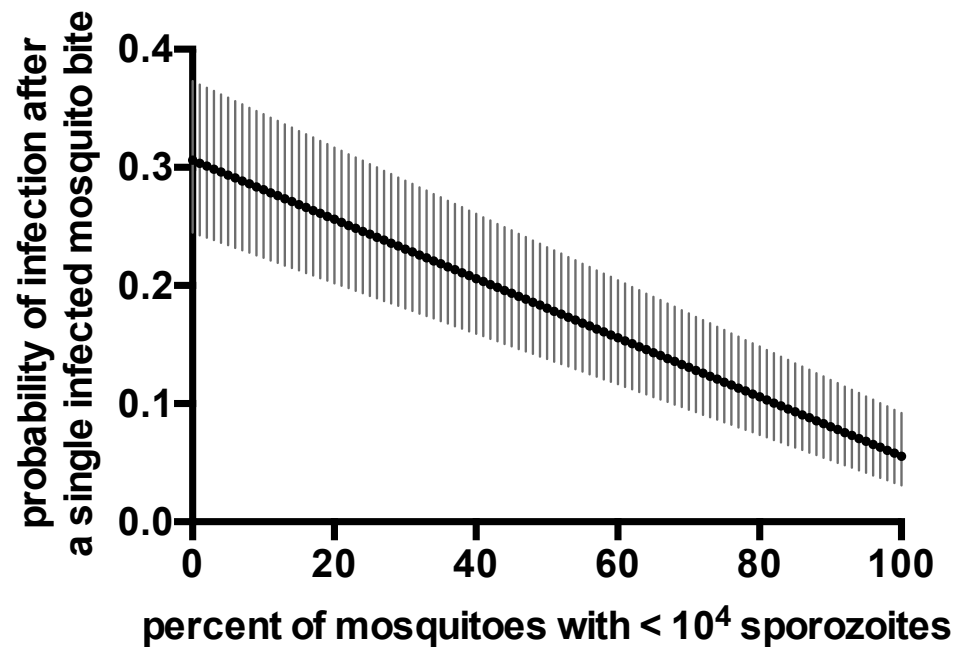

Supplement: S5 Fig — Shown is the decline in infection probability per mosquito bite as the fraction of mosquitoes with low (<10,000) sporozoite loads increases. In the full dataset of n = 412 mosquito feeds we found that probability of infection by mosquitoes with <10,000 sporozoite loads is b1 = 5.6% while bite by a mosquito with >104 sporozoites results in infection with probability b2 = 30.6%. If the proportion of mosquitoes with low (<10,000) sporozoite numbers is p, then the probability of infection per bite by a mosquito in such a population is given by b = p*b1+(1-p)*b2. Confidence intervals for these predictions were calculated using Jefferey’s intervals for binomial proportions for estimated values b1 and b2. (PDF) [file ppat.1008181.s005.pdf]
