# Supplementary material for: Experimental determination of the force of malaria infection reveals a non-linear relationship to mosquito sporozoite loads
Source: PLoS Pathog. 2020 May 26;16(5):e1008181. doi: 10.1371/journal.ppat.1008181 (PMC7295235; doi:10.1371/journal.ppat.1008181)
Supplement: Attachment — Submitted filename: PlosPathogens_ResponseToReviewers.pdf [file ppat.1008181.s007.pdf]

We thank the reviewers for their positive comments and thoughtful review of our paper. We address the questions and critiques below and have made edits and changes to the manuscript in response to the reviews that we feel make this a stronger paper.

Reviewer #1: The manuscript by Aleshnick and colleagues is a very timely, important and robust paper. The data are unique and provide important insights in developmental bottlenecks for malaria parasites. Whilst a non-human malaria model is used, it is plausible that the findings also have implications for Plasmodium species that are relevant for humans. I have only minor comments.

Reviewer #2: This manuscript investigated the impact of Plasmodium sporozoite load, blood-meal acquisition, probe-time, and probe location, on malaria infection probability. The results showed that mosquitoes with higher salivary gland sporozoites are more likely to initiate malaria infection, which provides reference data of a new useful phenotype for field studies. They also proved that infection probability was not impacted by whether a blood meal had been acquired by the mosquito. Overall, this paper will be interesting for readership working on malaria control and vaccine development.
However, some methods used in this study are not very appropriate or not well described. For example, the qPCR primers indicated in this paper are able to amplify three locations of the Plasmodium yoelii yoelii 17XNL genome, which was not mentioned anywhere in the manuscript.

Reviewer #3: This is an important manuscript which will have a strong impact on the field. In a tour-de-force experiments, the authors quantify the dynamics of Plasmodium transmission from mosquito to a mammalian host and reveal that only one fith of the infectious bites will result in malaria infections. The authors develop a model that explains non-linear relationships between the sporozoite loads in the mosquito salivary glands and transmission success, defining that malaria infections are transmitted by a small number of highly infected mosquitoes. These conclusions have important consequences for malaria epidemiology and for design of anti-sporozoite vaccine trials.
Although the number of sporozoites that are sufficient to initiate infection was not addressed in this manuscript, the authors provide strong evidence that only bites of mosquitoes that have more than 10,000 sporozoites will initiate the disease. Such quantitative approaches can be directly applied to the field studies instead of the currently used metrics of entomological innoculation rates that only consider the number of bites per person as a poxy for infectious bites.
Based on the novelty and significance of the presented data I strongly recommend this manuscipt for publication in PLoS Pathogens.

Thank you for the positive feedback on our study. We address the comment of Reviewer 2 on qPCR primers below.

Part II – Major Issues: Key Experiments Required for Acceptance

Reviewer #1: None.

Reviewer #2: 1. qPCR
There will be three amplification products using the qPCR primers indicated in this paper, according to the Plasmodium yoelii yoelii 17XNL genome (https://plasmodb.org/common/downloads/release-46/Pyoeliiyoelii17XNL/). The templates are contig AABL01000525, AABL01001425 and AABL01002193. Normally, genes with multiple copies in the genome are not good candidate reference genes for qPCR analysis, due to the instability of such kind of genes. This should be mentioned in the manuscript.

There are 4 sets of structurally distinct and differentially transcribed rRNA genes in all *Plasmodium* genomes (A, B, C, D types). Each set has an 18SRNA (SSU), an internal transcribed spacer 1 (ITS1), a 5.8S rRNA, a second ITS (ITS2), and the large-subunit (LSU) rRNA (or 28S rRNA). Each set is found on a different chromosome (Janse CJ, MBP 68:1994). Thus, there are four 18S genes and the C-type is found on chromosome 5. We are using primers specific for 18S C-type 18S rRNA, which is a single copy gene. However, there are regions that are identical among the 18S genes and so Reviewer 2's point is well-taken: Our primers could be binding to more than one of the 18S genes given the large regions of identity among the A, B, C, and D rRNA genes. Though they were designed to be C-type specific, we cannot be absolutely certain that this is the case because these genes have many regions of identity.

Due to the significant amount of identity among these genes, there is the complicating factor of the accuracy of the *P. yoelii* genome annotations. The Py17XNL genome was sequenced in 2002 and its annotation has not been updated. Two other Py genomes, Py17X and PyYM, have been sequenced more recently and the annotations of these genomes are more accurate. When we blasted our primer sequences and our amplified fragment sequence we obtained different results from each Py genome:

Py17XNL – We get one product with both primers, PY04657. However, we get two products with the forward primer alone, PY04657 and PY06474. Both products are misannotated as a hypothetical gene.

Py17X – We get two products, 070110 and 1249340, annotated as 18S rRNA genes but on chromosomes 7 and 12 which means they are amplifying the A and D 18S rRNA genes and not the C 18S rRNA gene, suggesting there is some mis-annotation in the genome.

PyYM – We get one product 0521740 18S rRNA on chromosome 5, which is the correct location for the C-type 18S rRNA gene.

We think that our primers are amplifying one product in the Py17XNL genome but cannot rule out that there is some amplification of one of the other 18S rRNA genes. However, it is important to note that there is no evidence that the rRNA genes are not stable: this is not a multi-gene family that undergoes recombination but rather a stable gene family in which different members are transcribed in different life cycle stages (Qi Y et al., mBio 6: e00117-15, 2015; van Spaendonk RML et al., MBP 105, 2000; van Spaendonk RML et al., JBC 276, 2001; Waters AP, Adv Parasitol 34, 1994).

Since sporozoite numbers are determined by comparison to DNA from a known number of sporozoites, even in the event that more than one member of this gene family is being amplified, this should not impact our results, since the DNA signal we are reporting is relative to a standard curve of known sporozoite numbers and this curve is itself verified by comparison to a plasmid standard curve.

Because of these considerations we think that a statement that our primers are amplifying multiple genes could be incorrect. We have added to the methods that we are amplifying the C-type 18S rRNA gene (Methods, page 29), however, we can add text about the identity of the different 18S rRNA genes and state that it is possible that our primers are amplifying other copies, i.e. the A, B or D genes of this family if Reviewer 2 thinks that is the best way forward. We are worried that this may be confusing to the reader, particularly because it is only a possibility and not a certainty, and in the end it we do not believe it impacts the quality of the data.

The authors were using known numbers of sporozoites to generate standard curve, in which way the DNA recovery rate should be able obtained, especially the authors also used 18s rRNA plasmid as standard (Supplementary Figure 1). It's better to include the DNA recovery rates for different numbers of sporozoites in the manuscript or in the Supplementary files.

We wish this were possible. We completely agree with the reviewer that this would be a better way, or at least a good back-up for sporozoite yield. However, sporozoites cannot be grown in vitro – we dissect mosquito salivary glands, homogenize them and use sporozoites in the homogenate for our standard curve. This genomic DNA prep is ~99% mosquito material so the DNA recovery rate is not indicative of how many sporozoites are in our sample. Because of issues such as these, we took a two-pronged approach to generating the standard curve – using both sporozoite number counted by hemocytometer and verifying this standard curve using a plasmid standard curve.

Method for measuring 18s rRNA plasmid copy numbers should also be included.

This was an omission in the original version of the paper and we thank Reviewer 2 for pointing this out. In brief we calculate plasmid size and the mass of a single plasmid molecule, then we multiply this by the copy number of interest and calculate the concentration of plasmid DNA needed to achieve the copy numbers of interest. We then prepare this solution (usually at 10^{10} copies in 4 microlitres) freeze aliquots and use one aliquot per experiment to perform serial dilutions of the plasmid. We have now added this to the Methods Section (page 29):

"Standard curves with known numbers of sporozoites were verified with a plasmid standard curve, using serial dilutions of a plasmid containing a fragment of the 18S rRNA C-type gene. This was performed by calculating the plasmid size and mass of a single plasmid molecule, multiplying this by the copy number of interest and finally calculating the concentration of plasmid DNA needed

to achieve the copy numbers of interest. We then prepared this solution (usually at 10^{10} copies in 4 microlitres) froze aliquots and used these to perform serial dilutions of the plasmid."

Also, a supplementary file/figure to show the primer amplification efficient and the standard curve would be useful for other people to re-use the data.

We agree. In Supplementary Figure 1B we show that the primers can efficiently and accurately amplify plasmid and sporozoites over 5 logs.

2. Controlled/uncontrolled probe time experiments
There were two mosquito feeding methods used in this study: controlled/uncontrolled probe time experiments. 1) In the controlled probe time experiments (page 29), some of the mosquitoes were forced to resume probing, which increased the probing by human manipulation. 2) Mosquitoes in the controlled probe time experiment groups also had more sporozoite load than in the controlled probe time experiment groups (Figure 3 legend). 3) The explore time of mosquito to mice were different in these two experiment groups: 30min for uncontrolled groups, while variety for the controlled groups. It seems there were multiple variances in these two experiments, should it be better to separate the analysis of these two experiments (Figure 2; page 8, parag 1; page 12, parag 2)?

The reviewer is correct, two different methodologies were used so that we could assess the impact of probe time on infection likelihood. For Figures 1, 3, 4, and 5, these two datasets were separated. However, since we found that acquisition of a blood meal had no impact on the likelihood of infection, we could include both datasets in the analysis of salivary gland load on infection likelihood. Importantly, Dr. Yenokyan, a Biostatician at Hopkins and co-author of this study, included robust variance estimates in all analyses to adjust for potential within-experiment correlations of malaria infections (see Statistical Analyses in the Methods Section of the paper). Thus, it was statistically appropriate to include both datasets in the infection likelihood analysis.

Nonetheless, the Reviewer raises an interesting point and we analyzed the data to see if the findings shown in Figure 2 held if the analyses were performed with the separated datasets.

Logistic Regression:
Analysis of the separated datasets to calculate the odds ratio of malaria infection when mosquitoes have ≥ 20,000 salivary gland sporozoites was as follows:
Controlled Probe Time data alone: Odds Ratio of malaria is 8.45 (95% CI 3.85 -18.54).
Uncontrolled Probe Time data alone: Odds ratio of malaria is 7.52 (95% CI 1.99 - 28.27).

Mathematical Modelling:
We redid the modeling on the separated datasets:
(A) Controlled Probe Time (B) Uncontrolled Probe Time

As shown below, the Threshold model fits all datasets better than the other two models, as we showed in the entire dataset. When we performed this analysis we did not fix the threshold in fitting the model to the data. Not fixing this parameter gives more robust comparisons, since a priori we do not know the exact value of the threshold. When we redid this analysis on the entire dataset, we realized that in original analysis the threshold (S*) was fixed at 20,000 salivary gland sporozoites. We have now performed the analysis in Figure 2C without an a priori determined threshold (S*) and with this more robust analysis, the *w* (Akaike weight) is lower for the Threshold model than it was in our original analysis where. The new Akaike weights shown in the revised Figure 2C are: Threshold 0.77, Powerlaw 0.23, Single Hit 0.00. We thank Reviewer 2 for prompting us to reanalyze these data: the results with the separated datasets reflect our original finding and the new analysis, where S* is not fixed, is more accurate.

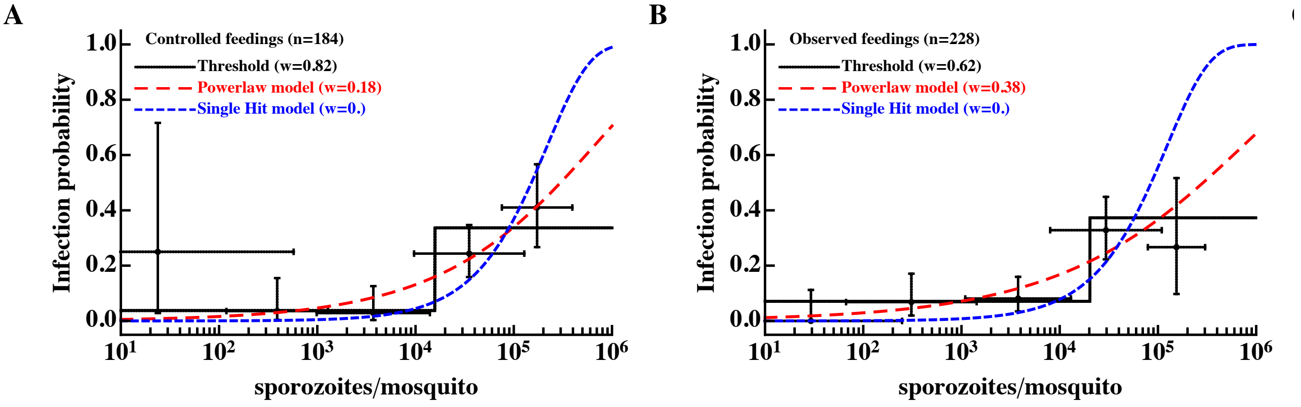

Reviewer #3: I do not think that additional experiments are required.

Part III – Minor Issues: Editorial and Data Presentation Modifications

All 3 Reviewers raised concerns about the way in which the probe time data were presented and here we address these comments together. We agree that our description of the impact of probe time on infection was confusing. This is because we were trying to compare two very different datasets: the uncontrolled probe time experiments and the controlled probe time dataset. There are significant differences between these two datasets, i.e. the controlled PT consists of 120 mouse-mosquito pairs with probe times of 10 sec or 1 min while the uncontrolled PT has only 18 mouse-mosquito pairs with probe times ≤ 1 minute and 203 mosquito-mouse pairs with probe times >1minute. We used the uncontrolled probe time data to put forward the hypothesis that at probe times over 1 min (the majority of the uncontrolled probe time dataset) we could not discern any difference in infection likelihood with increasing probe times. However, the uncontrolled probe time experiments were not designed to test probe time effects and the 203 uncontrolled probe

times are distributed across a spectrum of probe times ranging from 61 to 2536 seconds. The frequency of distribution of the controlled and uncontrolled probe times is completely different as can be seen in the plot below.

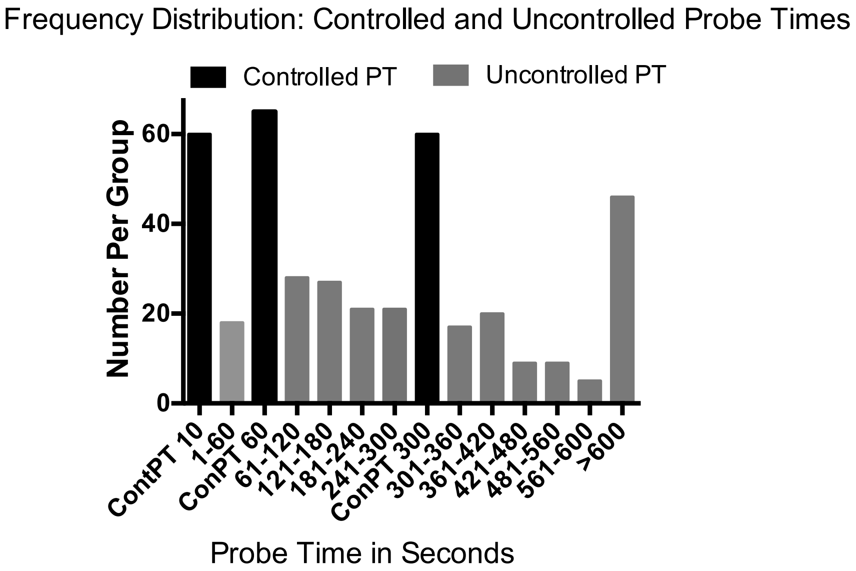

Regarding the uncontrolled probe time data, the lack of discrete probe time groups which would give a larger "n" for comparisons, and the large distribution of probe times, preclude us from using these data to make definitive statements about probe time and infection. Since it is clear from our controlled PT data that there is a statistically significant relationship between probe time and infection likelihood, we decided to limit our discussion on probe time to the controlled probe time dataset, where the experiments were specifically designed to test the relationship between probe time and infection.

We have therefore removed Figure 3B from the paper and removed the text, in the Results and Discussion, of these data.

Reviewer 1

Fig3b is confusing. Too much attention is drawn to the first bar with a very low number of observations. It would be more appropriate to combine the two lower bins

The reason we separated out the ≤10 second bin in Fig 3B was to make the lack of comparability between the uncontrolled and controlled PT data more clear, i.e. while the controlled PT experiments had large numbers of mosquitoes with short and moderate probe times, the uncontrolled PT data did not. However, after reading the comments from all 3 reviewers we came to the conclusion that the uncontrolled PT dataset does not reveal much about the impact of probe time on infection likelihood because there are so few mosquitoes that probed for ≤1 minute in this dataset (n=18), compared to the 120 data points in the controlled probe time dataset. We therefore decided to remove Figure 3B and focus on the experiments in which probe time was specifically tested as a variable.

Reviewer 2

Figure 3: The authors mentioned that spz load may explain the different patterns in panel A and B. An extra scatter/box diagram of the spz load in different bin would help to clarify.

We apologize for the confusion. Actually we do not think that sporozoite loads are responsible for the different patterns in 3A and 3B. What we were trying to communicate, was that sporozoite loads likely explain the difference in the overall infection likelihood between the 3A and 3B datasets. Nonetheless, we do understand how our statement at the end of the legend for Fig 3 could be confusing. By removing Fig 3B the probe time analysis is now limited to experiments in which the salivary gland loads are comparable among the different experimental groups.

Panel A, how many samples in the 5min group have been forced to resume probing?

We think Reviewer 2 is referring to interruption of blood feeding. In the 5 min probe time experiments, a single mosquito in a small plexiglass cage was placed on the ear and probe time was recorded. There were 3 possible outcomes and these are all discussed in the Methods Section: 1) The mosquito did not probe for 5 min even though the mouse was exposed to the mosquito for up to 30 min. This happened 3 times and these mosquito-mouse pairs were removed from the analysis. 2) The mosquito probed for a total of 5 min without finding blood. These probes were either in one continuous probe (rarely) or separated in time (i.e. the mosquito withdrew its mouthparts and either began probing in an adjacent location, or flew away, landed and reinitiated probing in the same or a different location). 3) In the course of probing, and prior to the 5 min time point, the mosquito found blood. In this case the cage with the mosquito was immediately lifted from the mouse to halt blood feeding and gently placed it back down on the ear. There are some parameters of the probing events that we did not record, which in hindsight may have provided additional insight into the factors leading to disease risk; the percentage of mosquitoes in groups 2 and 3 above is something we should have recorded. However, whether sporozoite regeneration in the salivary duct is continuous throughout mosquito probing or takes place at some time distinct from the probing event (a question we are interested in but is not in the scope of this paper) we believe that short breaks in probing, whether to dislodge a feeding mosquito or encourage it to resume probing, likely do not impact disease probability, which was the outcome these studies were designed to address.

What's the significant level between 10 sec and 1min group?
Thank you for pointing out this omission. The difference in infection likelihood between the 10 sec and 1 min groups was not significant. We have now included this result in the Figure and Figure Legend.

panel B, there was no mouse infected by mosquito (more than 100 samples) with >5min probing, any reason?

The data in Figure 3B show that 16% of mice became infected with >5 to 10 min probing and 15% of mice became infected with >10 minutes of probing. We are not exactly sure what the reviewer is referring to but please let us know if there is something that doesn't make sense! Nonetheless for the reasons stated above we have now removed Figure 3B from the paper.

The spz loads between ">5-10 min" and ">1-5 min" were not significantly different while the infection rates were quite different (page 15), why?

The infection rates for mice probed upon for >1-5 minutes was 10% and for those probed upon for >5-10 was 16%. The difference in infection likelihood between these groups was not significant though we agree that there is a trend, which we think is because of the increased probe time. Nonetheless for the reasons stated above we have now removed the uncontrolled probe time data from this figure.

Page 15: does "11,766 +/- 16,786" mean "range from 11,766 to 16,786"?
We apologize for the confusion and should have specified that this was standard deviation. However, these data are now removed so this should not be an issue.

Reviewer 3
I was also confused by the discussion of the results presented in Figure 3A: "As shown in Figure 3A, mosquitoes that were allowed to probe longer were more likely to initiate a malaria infection (p=0.020, Fisher's exact test) with pairwise comparisons showing a statistically significant difference in infection probability between the 10 sec and 5 min probe times (p=0.021) and a less robust but significant difference between the 1 min and 5 min probe times (p=0.025). The difference between the 1 min and 5 min groups was not significant by non-parametric tests such as the Mann-Whitney (p=0.15)."
Could the authors use the appropriate statistical tools? If the data is normally distributed, the use of parametric tests is justified. If the data is not normally distributed, only non-parametric tests should be used. The authors have to make clear whether the observed differences are statistically significant or not and show variability and p-values on the graph.

We agree. In the original version of the paper we added the Mann-Whitney analysis in order to tie into the uncontrolled probe time data. We no longer include the uncontrolled probe time data and outline below the statistical analyses we performed. This information is now included in the Legend for Figure 3 and in the Methods (pg. 32).

The Fisher's exact test was used to test whether there is a significant overall relationship between probe time and malaria infection (p=0.020). Following this we performed pairwise comparisons using logistic regression analysis where malaria infection was the dependent variable and probe time was the covariat, represented by 2 indicator variables (1 min and 5 min probe time groups) with the 10 sec probe time group being the reference. The p-value for the slope of each group represents the comparison of that group to the reference. Comparison of the 2 slopes gives the p-value for the 1 min versus the 5 min groups.

Pairwise comparisons show a statistically significant relationship between the 10 sec and 5 min groups, and the 1 min and 5 min groups. There is not a significant difference between the 10 sec and 1 min groups. We have added the results of all of the pairwise comparisons to Figure 3 and included p-values for each pairwise comparison in the legend.

The Figure 3B is also confusing. If the authors identify problems in the dataset, it should not be presented in the main figure as such. Instead, the authors could only present the data for the groups that can be compared (for example, the group probing for 1-5 min and the group probing for 5-10 min).

We agree. Above we have outlined our reasons for deleting Fig 3B which are largely in line with the issues raised by Reviewer 3.

Reviewer #1: Abstract: 'being 7.5 times more likely to initiate a malaria infection' suggests a precision that is not supported by the data. I would suggest to rephrase this and make it more descriptive (e.g. 'considerably more likely… compared to mosquitoes with lower infection burden'

This is now changed this to "significantly" in the abstract and author summary.

Author summary: 'In this study, using a rodent… results in malaria infection' contains a duplication of messages. Minority infecting and majority non-infecting is clearly the same. I would suggest to simplify this.

Agree, the sentence now reads, *"In this study, using a rodent model of malaria, we found that the majority of infective bites do not result in malaria infection."*

I would strengthen the author summary by making not only a comment on the importance for interventions but also for understanding the epidemiology of malaria.

We have now changed the last sentence of the author summary to read, *"Overall this work contributes to our understanding of the epidemiology of malaria and should aid the development of malaria elimination strategies."*

The introduction is very well written and nicely illustrates (very old) literature and current-day relevance of the question addressed.

We thank this reviewer for their appreciation of our effort to situate this work in a larger context.

The first section of the results explains why P. yoelii was chosen. Part of the argument is missing. I believe that the authors want to say that because yoelii has such a high likelihood of resulting in detectable infections, it provides a sensitive system to detect/quantify potentially infectious bites. It would be good to mention this specifically.

We agree with the reviewer and thank you for the suggestion. The first two sentences of the Results section now read:

"The rodent malaria parasite P. yoelii was used for these experiments because it provides a sensitive system to detect potentially infectious bites. Indeed, P. yoelii sporozoite infectivity for laboratory mice is similar to the infectivity of human and primate malaria sporozoites for their natural hosts [12-16]."

The direct observation of effective contact of mosquitoes is a considerable strength of the paper

Thank-you for this comment.

Figure 1 and 5b should come with error bars
We have now added error bars. Since these figures show a binary readout, i.e. infection (Fig 1) or blood meal acquisition (Fig 5B), we generated error bars by calculating 95% confidence intervals calculated using Jefferey's intervals for binomial distribution. This is stated in the figure legends.

Page 8, 'over 400': give exact number.

We have now changed this to "412".

Figure 2c. the step appears completely driven by one data point that lies well above the fitted line. It would be good to discuss this in the discussion. The data overall are very convincing but the strong claim on this step is a weaker part of the (overall very strong) paper. I am unconvinced the step would be observed again if the study would be repeated.

The data points in Figure 2C are averages, with each point being the average of 41 data points (mosquito-mouse pairs), except the last point which is the average of 43 mosquito-mouse pairs. These data "aggregates" were included only to help the reader visualize the data because it is difficult to see patterns in data that are binary (0 = no infection; 1= infection). The actual mathematical modeling was performed on entire raw dataset (n=412) and not the averaged data. The fit of these three models is compared using Akaike weights, with larger "w" signifying a better fit of the model to the data. The threshold model fits the data somewhat better (w=0.77) than the continuous models, powerlaw (w=0.23) and single hit (w=0.00).

Nonetheless, the reviewer raises a point that we had given some thought to as we were writing the paper. Namely, is this a "hard step" or a more gradual "softer step" that one might expect with a biological process. To look at this in more detail we fit several models, all of which incorporated a rapid increase in infection likelihood between gland loads of 10,000 to 20,000. We show the results of these analyses in Figure S3 and discuss this in the last paragraph on page 11. In summary, all models that incorporate a rapid change in infection probability between 10,000 to 20,000 salivary gland sporozoites fit the data relatively well (based on the Hosmer-Lemeshow test) and models that had a "softer"

threshold fit the data marginally better than the “hard” threshold/step model (based on Akaike weights).

For clarity, we made some changes in the legend describing Figure 2C to better distinguish the lines, which reflect the modeling, from the data points, which are shown for illustrative purposes:

(C) Mathematical modeling of infection probability as it relates to salivary gland sporozoite load. *Three alternative models, (single hit, powerlaw, and threshold), were fit to the entire raw dataset (n=412) and compared using Akaike Information Criterion to generate Akaike weights (w). The threshold model best describes the probability of malaria infection as a function of salivary gland sporozoite number (highest weight). The data points on the graph are shown for illustrative purposes: Raw data were binned with equal number of mosquitoes in each group (n=41), except the last group, which has 43 mosquitoes. Error bars on the y-axis show 95% confidence intervals calculated using Jefferey’s intervals for binomial distribution [66] and on the x-axis show 67% confidence intervals calculated using normal distribution. The single hit, powerlaw, and threshold models are described in the Materials and Methods by equations 1, 2, and 3, respectively) and fit was determined using the maximum likelihood method (equation 6 in Materials and Methods).*

Methods are great and very complete; the discussion is appropriate and well written. It is great to see the Ross McDonald model being criticized in a positive and data-supported manner.

We thank the reviewer for this feedback.

Reviewer #2: Page2, Abstract: “sporozoites to patent blood-stage infection”, patient?

Patent is the correct word – it means recognizable, i.e. by a blood smear we can see parasites.

Page 6, Supplemental Table. An extra column will be much helpful to clarify to the experimental methods, other than by probe time values of (10, 60, and 300 seconds). Maybe also include Probe time (s) for mosquitoes without infection (Salivary Gland Load = 0).

All probe time, probe location, blood meal acquisition, salivary gland load, and malaria infection data are in the table. Since its in an excel sheet, the reader can organize and sort the data according to any of these parameters, not just probe time. There is also a paragraph accompanying the table (on the right hand side of the excel sheet), explaining the experimental methodology, with more details being in the Methods section. In that paragraph we state “SMFE where the probe time was controlled are indicated by discreet probe time values of (10, 60, and 300 seconds)”. This should enable the reader to distinguish controlled and uncontrolled probe time experiments. SMFE: single mosquito feed experiment

In the methods section we state that mosquitoes without salivary gland sporozoites and their accompanying mouse were removed from the study since the purpose of this study was to determine how likely an infected mosquito is to initiate a malaria infection. There were not sufficient numbers of uninfected mosquitoes to do statistics on whether infection impacted probe time. While we agree with the reviewer that this is an important question, other groups have specifically designed studies to test this, and as those papers had much higher numbers of uninfected mosquitoes we think it best to defer to those studies (Rossignol PA, et al., Am J Trop Med Hyg, 1984; Anderson RA et al., Proc Biol Sci, 1999; Wekesa JW et al., Am J Trop Med Hyg, 1992).

Page 8 parag 2 & page 23 parag 2: what's the range of sporozoite load in field samples?
We could only find 6 papers that measured salivary gland loads of infected mosquitoes in the field and these are all discussed and cited in the manuscript. The ranges of sporozoite loads in these papers are:
Beier 1991: An. gambaie 2-117,544 and An. funestus 5-41,830
Beier 1997: 125 to 79,875
Burkot 1987: 150-10,000
Pringle 1966: An. gambaie 130-245,760 and An. funestus 82-77,270
Shiff 1998: range not given
Shute 1965: An. funestus 370-45,450 and An. gambaie 135-68,200

It is difficult to succinctly summarize the data from these papers for our readers because each study reports their results differently. Since our findings suggest that what matters is whether the salivary gland sporozoite density is >10,000 in our discussion we focused on the proportion of mosquitoes in the field samples that had >10,000 sporozoites.

Page 8 second last lane: add reference.
Thank-you, this is now included. Its reference 30.

Page 9, page 12: The abbreviation for p value should be unified.
Thank-you for noting this. We've gone through and made sure that all p values are in lower case not italicized.

Page 12 & Figure 2: the infection probability seems to reach a plateau after 20,000 spz load, any possible explanations?

We agree and also note that infection likelihood plateaus. We address this in the Discussion, on page 23:
"While we do not understand why highly infected mosquitoes are not capable of initiating infection 100% of the time, several investigators have observed that highly infected mosquitoes inconsistently inoculate large numbers of sporozoites [22, 30, 32]. Indeed, in a previous study we found that the subset of highly infected mosquitoes inoculated >100 sporozoites about 40% of the time [22], a finding that is consistent with the frequency with

which these mosquitoes initiate infection, suggesting that a threshold number of ~100 sporozoites may be required to reliably initiate infection."

There were multiple logistic regressions mentioned in this manuscript, a summary of the parameters in these regressions should make the results much clearer.

We agree and have summarized these parameters in the Methods Section on pages 31-34. All equations are listed and equations 8 & 10 explicitly state the parameters. Further, we refer to each equation in the text and figure legends when the graph it generated is displayed.

Figure 4: legend for pie chart.
We agree and have changed the legend for Figure 4 to make this more explicit.

Figure 5: it's better to mention that it's based on 30min of mosquito-mouse exposure experiment.
While we agree with the reviewer, we feel that it could be confusing for the following reason: Though we allowed each mosquito 30 minutes of access to its mouse, in the two cases where mosquitoes were still probing at 30 minutes, we allowed them to continue until they desisted on their own. Thus there are 2 mosquitoes probed for 2059 and 2536 seconds, and these can be easily observed in Fig 5A. For this reason, we prefer to leave it as is with the explanation in the methods on page 27:

"Mosquitoes were given access to mice for 30 minutes, though in 2 cases, mosquitoes that were still probing at 30 minutes were allowed to continue probing until they desisted."

Any differences in probe time and spz load, between mosquitoes that able to get a blood-meal and not able to get blood-meal in 30min?

Good questions. We observed that salivary gland load had an impact on the likelihood of obtaining a blood meal (see Figure 5C). We also observed that probe time was decreased in those mosquitoes that obtained a blood meal (see below, $p<0.0001$). However, because success in blood meal acquisition would be expected to be associated with a decrease in probe time, i.e. once mosquitoes find blood they take it and leave, we decided not to include these data in the paper, focusing instead on the impact of the parameters we measured on infection likelihood and on blood meal acquisition likelihood. Nonetheless we agree that there are many interesting things one could ask with our dataset and this is why we included all of our data with the paper.

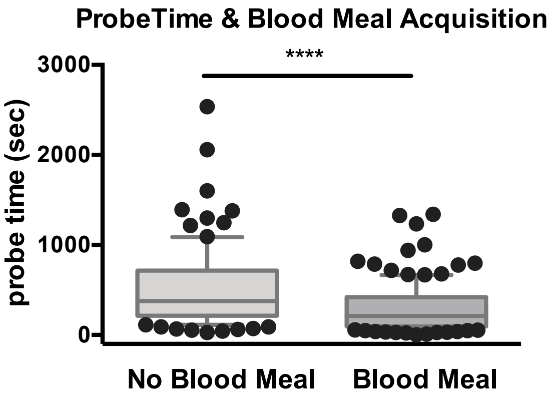

Page 21 lane 4: what's the proportion in this study and in the field?

The proportion of highly infected mosquitoes, i.e. >10,000 salivary gland sporozoites, was ~50%. This is shown in Supplemental Figure 1 and stated in the legend to this figure, *"216 mosquitoes had <10,000 and 196 mosquitoes had >10,000 salivary gland sporozoites."*. It is also shown in Figure 2A (numbers above each bar) and these data are in the Supplemental Table. In the field it varies from one study to another: Its 12% in Beier (1991); 17% in Beier (1997); 5% have >9000 sporozoites in Shiff (1998), and 25% have >16,000 in Pringle (1996). Thus, we could not be specific and decided to keep this statement more general. On page 23 we state: *"Quantification of salivary gland sporozoite loads in wild-caught mosquitoes by investigators working in different regions of sub-Saharan Africa found that the percentage of infected mosquitoes with sporozoites loads over 10,000 ranges from 5-25%, with most studies putting this number at ~10% [30, 41-43, 51, 52]."*

Page 24: what's the infection probabilities estimated from field studies?

This is a poorly understood number in the field, which is why we undertook this study. This is the topic of the first paragraph of the Discussion where we state, *"Thus, a proportional factor "b" was added to EIR (Box 1) where "b" is the fraction of infected human-feeding mosquitoes that initiate a malaria infection. Though "b" cannot be directly determined in humans, several studies have compared the incidence rate of malaria in infants to the biting rate of infected mosquitoes to arrive at estimates of "b" that range from 1 to 10% [7, 10, 11]."* If our explanation in the Discussion section of the paper is not clear, please let us know how we can clarify.

Reviewer #3: To increase the clarity of the manuscript I would like the authors to restructure the information split between the Introduction and Discussion. Ross-MacDonald formula should be described in the Introduction to setup the stage for the current study. otherwise, the information appears in both places but is very imprecise in the Introduction which is cryptic for a wide audience of readers.

We understand what the Reviewer is saying. In part we set it up this way to keep the Introduction short and keep the reader interested. We are open to adding the Ross-MacDonald formula to the Introduction, however are hesitant because of Reviewer 1's statement *"The introduction is very well written and nicely illustrates (very old) literature and current-day relevance of the question addressed."*

Eliminating repetitions between the Results and Discussion will also make the stronger the Discussion part.

We agree and have gone through the Results and Discussion to eliminate repetitions.

On page 6 where we discuss the rationale for looking at the impact of blood meal acquisition on malaria infection likelihood, the text was redundant with the Discussion and we have deleted the text below from the Result section:
Importantly, volunteers in human malaria vaccine trials are typically challenged by five infected mosquito bites, where "bite" is understood to mean that the mosquito has blood fed. Thus, mosquitoes that probe but do not imbibe blood are replaced until a total of five have imbibed blood, resulting in a wide range of exposure to infected mosquitoes among the volunteers [25-27].

In the results section pertaining to location of feeding we left in the 2 sentences of 'discussion-like' text because we do not discuss these findings in the Discussion.

With the removal of the uncontrolled probe time data we also removed the discussion-like portions of these data from the Results section.
